# Contractile Effects of Semaglutide in the Human Atrium

**DOI:** 10.3390/pharmaceutics16091139

**Published:** 2024-08-28

**Authors:** Joachim Neumann, Katarína Hadová, Jan Klimas, Britt Hofmann, Ulrich Gergs

**Affiliations:** 1 Institute for Pharmacology and Toxicology, Medical Faculty, Martin Luther University Halle-Wittenberg, Magdeburger Straße 4, D-06112 Halle (Saale), Germany; ulrich.gergs@medizin.uni-halle.de; 2 Department of Pharmacology and Toxicology, Faculty of Pharmacy, Comenius University, SK-83232 Bratislava, Slovakia; hadova@fpharm.uniba.sk (K.H.); klimas1@uniba.sk (J.K.); 3 Department of Cardiac Surgery, Mid-German Heart Center, University Hospital Halle, Ernst-Grube-Straße 40, D-06097 Halle (Saale), Germany; britt.hofmann@uk-halle.de

**Keywords:** semaglutide, GLP-1 receptor, human atrium, inotropy

## Abstract

Semaglutide is a glucagon-like peptide 1 receptor (GLP-1R) agonist. GLP-1R agonists are used to treat type 2 diabetes and obesity. It is currently unknown whether semaglutide can directly increase force of contraction (FOC) in the human heart. We tested the hypothesis that semaglutide might increase the FOC in the isolated human atrium. To this end, we conducted contraction experiments in isolated human right atrial muscle preparations (HAP). HAP were obtained during open-heart surgery. We detected a concentration- and time-dependent positive inotropic effect (PIE) of semaglutide in HAP. These PIEs were accompanied by increases in the rates of tension development and tension relaxation and a reduction in muscle relaxation time. The PIE of semaglutide in HAP was attenuated by H89, an inhibitor of the cyclic AMP-dependent protein kinase and by ryanodine, an inhibitor of sarcoplasmic Ca^2+^ release. Semaglutide up to 100 nM failed to exert a PIE in isolated electrically paced (1 Hz) wild-type mouse left atrial preparations studied for comparison. Our data suggest that semaglutide can increase the FOC in the atria of patients at therapeutic drug concentrations.

## 1. Introduction

Type 2 diabetes mellitus and obesity are risk factors for cardiac diseases like hypertension, cardiac hypertrophy and heart failure. Hence, the treatment of type 2 diabetes and obesity with novel drugs is gaining more and more clinical attention to prevent or at least treat heart failure. One class of drugs that have shown promise in clinical trials against type 2 diabetes are the so-called glucagon-like peptide 1 receptor (GLP-1R) agonists. They mimic the effect of the endogenous peptide GLP-1. GLP-1 is formed in specialized cells in the gut. Upon oral intake of glucose or fat rich meals, GLP-1 is released from the gut and reaches its target organ in the human body. GLP-1 acts via GLP-1 receptors and via these receptors can increase in the pancreas the release of insulin and inhibit the release of glucagon. In sum, the glucose level is reduced and thus type 2 diabetes can be treated. Hence, exogenous compounds were developed to mimic the action on GLP-1 receptors. The first drug was exenatide, structurally very distant in sequence from GLP-1. But exenatide was able to treat type 2 diabetes. One drawback of exenatide was its weak efficacy in reducing body weight. The reduction in body weight is probably due to the stimulation of brain GLP-1R that leads to satiety. Thus, more effective drugs to reduce body weight have emerged that are of interest in the present study, namely liraglutide and semaglutide [1].

**Figure 1:**
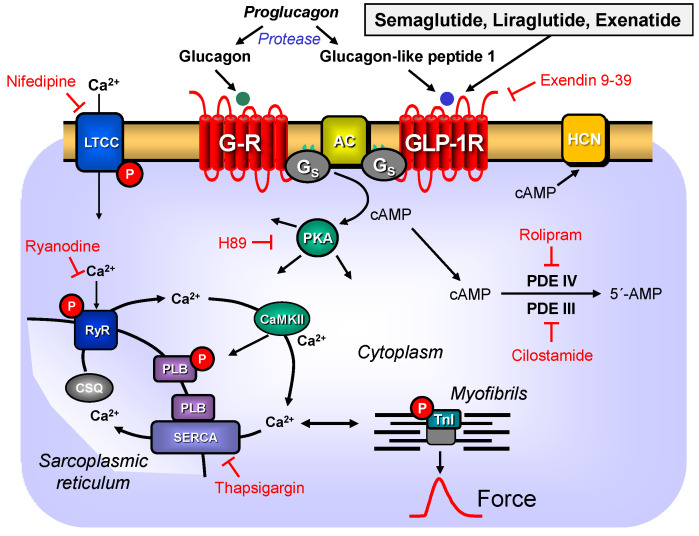
Mechanism(s) of action of GLP-1R in cardiomyocytes: Proglucagon is cleaved by a protease to glucagon-like peptide 1. This binds to the sarcolemmal glucagon-like peptide 1 receptor (GLP-1R) and can be antagonized by exendin(9-39). Via stimulatory GTP-binding proteins (Gs), this activates the adenylyl cyclases (AC) to form cAMP that then activates cAMP-dependent protein kinases (PKA). PKA (inhibited by H89) phosphorylates and, e.g., activates L-type-Ca^2+^-channels (LTCC, inhibited by nifedipine), the ryanodine receptor (RyR, inhibited by ryanodine), phospholamban (PLB) and the inhibitory subunit of troponin (TnI). PLB inhibits the sarco(endo)plasmic reticulum Ca^2+^ ATPase (SERCA) (which is inhibited by thapsigargin) and cAMP can directly activate hyperpolarization-activated cyclic nucleotide-gated channels (HCN) that increase the beating rate in sinus node cells. cAMP is degraded by phosphodiesterases (PDE). Glucagon is an agonist at glucagon receptors (G-R) and can couple like the GLP-1R via stimulatory Gs proteins and thereby glucagon can activate the adenylyl cyclases.

The GLP-1R (Figure 1)-like β-adrenoceptors couple via stimulatory G-proteins. Then, adenylyl cyclases increase intracellular cAMP levels [1]. Such an increase in cAMP levels is expected to increase via cAMP-dependent protein kinases (PKA) the phosphorylation state and the activity of regulatory proteins like phospholamban (Figure 1). Finally, the force of contraction (FOC) and the rate of relaxation should increase, and the time of relaxation should decrease like with the β-adrenoceptor agonist isoprenaline. Like isoprenaline, in the heart semaglutide and liraglutide should increase the FOC and the beating rate. In human atrial preparations (HAP), exenatide stimulated the FOC, but whether exenatide increased the rate of tension development or relaxation time or time to peak tension in HAP was not reported [2].

The positive inotropic effects (PIEs) of exenatide were accompanied by and probably caused by an increased phosphorylation state of regulatory proteins like phospholamban in the HAP [2]. The PIEs of exenatide were also blocked by H89 (Figure 1), an inhibitor of the activity of cAMP-dependent protein kinase and by exendin(9-39), a proteolytic fragment formed from exenatide and an antagonist at the GLP-1R [2].

Moreover, exenatide, liraglutide and semaglutide have been tested in preclinical models of obesity and heart failure. Typically, these GLP-1R agonists conveyed protection against cardiac hypertrophy in chronic experiments. In acute experiments, GLP-1 agonists reduced damage to the heart from ischemia in wild-type mice compared to mice with knock out of GLP-1R. Hence, one can ask whether mice are a good model for the human heart in this regard. In other words, do exenatide, semaglutide and liraglutide exert positive inotropic effects and perhaps also positive chronotropic effects in the heart that are qualitatively similar to the effects of GLP-1R in the human heart. As models for the present study, we decided to use left and right atrial preparations from wild-type mice and right atria preparations from human hearts in order to provide comparability.

Exenatide (3 nM, 5 nM) failed to alter left ventricular pressure in isolated perfused hearts from adult mice [3,4]. However, 0.3 nM GLP-1 increased left ventricular pressure in isolated perfused hearts from adult mice [4]. This increase in left ventricular pressure was lacking in mice with deletion of the GLP-1R: this implies that GLP-1 can increase FOC via GLP-1 in the adult mouse heart [4].

Hence, we tested the hypothesis that semaglutide, liraglutide and exenatide might increase the FOC in the human atrium and left or right atrial preparations of mice. To this end, we performed contraction experiments with isolated muscle preparations. A progress report has been published as a congress abstract [5].

## 2. Materials and Methods

### 2.1. Contractile Studies in Mice

The breeding, housekeeping and handling of the mice was in line with local animal protection requirements. The right or left atrial preparations from wild-type mice (CD-1, random sex, about 100 days old) were isolated and mounted in organ baths, as previously described [6]. The bathing solution of the organ baths contained a modified Tyrode’s solution: 119.8 mM NaCI, 5.4 mM KCI, 1.8 mM CaCl_2_, 1.05 mM MgCl_2_, 0.42 mM NaH_2_PO_4_, 22.6 mM NaHCO_3_, 0.05 mM Na_2_EDTA, 0.28 mM ascorbic acid and 5.05 mM glucose. The solution was continuously gassed with 95% O_2_ and 5% CO_2_ and maintained at 37 °C and pH 7.4 [6,7]. Spontaneously beating right atrial preparations from mice were used to study chronotropic effects and inotropic effects. Electrically stimulated (1 Hz) left atrial preparations from mice were only used to study inotropic effects. The FOC was quantified in electrically paced isolated atrial preparations under isometric conditions. The duration of electrical stimulation with a rectangular impulse of direct current lasted for 5 milliseconds. The voltage was 10% higher than necessary to initiate contraction. Muscles were stretched such that the maximum basal force was generated and then allowed to stabilize for 30 min before drug application started. Isometric contraction data were recorded using a PowerLab system (ADInstruments, Oxford, UK). Subsequently, the data obtained from the isometric contractions were processed using the LabChart Pro V8 software (ADIstruments, Oxford, UK). Several different protocols for drug application were used as follows. After equilibration was reached, either exenatide (n = 5) or semaglutide (n = 28) or liraglutide (n = 5) was cumulatively added to left atrial or right atrial preparations to establish concentration–response curves. Thereafter, glucagon and then isoprenaline were added.

### 2.2. Contractile Studies on Human Preparations

This study in patients complies with the Declaration of Helsinki and has been approved by the local ethics committee. Informed written consent was obtained from all patients included in the study. Contractile studies on HAP were performed with the same setup and buffer used in the mouse studies above. The FOC was quantified in electrically paced isolated right atrial preparations. The duration of electrical stimulation with a rectangular impulse of direct current lasted for 5 milliseconds. The voltage was 10% higher than necessary to initiate contraction. The muscles were stretched to produce maximum baseline force and then allowed to stabilize for 30 min before drug administration began. The samples were obtained from 10 male patients and 4 female patients, aged 45–83 years (mean ± SD: 70.3 ± 8.4 years). The patients suffered from coronary diseases (two and three vessel diseases). Cardiovascular drug therapy included metoprolol, furosemide, apixaban, statins and acetylsalicylic acid. All patients receiving GLP-1R agonist therapy were excluded from the study because receptor expression and/or sensitivity might be altered. From each patient sample, approximately four to eight trabeculae were isolated, and of these isolated atrial preparations, 40 were of sufficient quality for experimentation. The methods used for atrial contraction studies in human samples have been previously published and were not altered in this study (e.g., [8,9]). The drug application was as follows: after equilibration was reached, semaglutide, liraglutide or exenatide were added cumulatively. In several experiments, semaglutide was applied at a single concentration to measure the effects of ryanodine to block ryanodine receptors, or H89 to block protein kinase A, or nifedipine to block L-type Ca^2+^ channels, or thapsigargin to block SERCA. Finally, isoprenaline was added to stimulate FOC maximally. In other experiments, semaglutide was cumulatively applied, then exenatide and thereafter exendin(9-39) [10] was given.

### 2.3. Detection of Messenger Ribonucleic Acid (mRNA)

Frozen samples of human right atria were homogenized mechanically in liquid nitrogen and subsequently subjected to acid phenol–guanidinium thiocyanate–chloroform extraction (TRI Reagent^®^, Sigma-Aldrich, St. Louis, MO, USA). The RNA extraction was performed according to the instructions of the manufacturer. RNA was subsequently precipitated from aqueous phase by isopropanol and after centrifugation the RNA pellets were washed twice with ethanol. The samples were tested by electrophoresis in 2% agarose gel (Agarose, Sigma-Aldrich, USA) for RNA quality. Intact RNA samples were reverse transcribed into the complementary DNA (cDNA) structure using High-capacity cDNA Reverse Transcription Kit with RNAse inhibitors (Applied Biosystems, Grand Island, NY, USA). We performed quantitative real-time PCR (RT-qPCR) analysis with the use of a StepOnePlus™ Real-Time PCR System (Thermo Fisher Scientific, Waltham, MA, USA) with SYBR™ Select Master Mix (Thermo Fisher Scientific, USA). The expression of glucagon-like peptide receptor 1 was measured using gene-specific primers (forward: TCCTCCTCGGCTTCAGACAC, reverse: TCGCAGGATGAAGGATGCAA). The primer sequences were designed using the tool Primer-BLAST [11]. Glycerin aldehyde phosphate dehydrogenase (GAPDH) was used as housekeeping gene.

### 2.4. Data Analysis

Data shown are the means ± standard error of the mean. Statistical significance was estimated with Graphpad Prism 9 (GraphPad Software, Boston, MA, USA) using the two-way analysis of variance (ANOVA: mixed-effects model with Geisser–Greenhouse correction and Dunnett’s multiple comparisons test). A *p*-value < 0.05 was regarded as significant. The Prism software was also used for the visualization of data.

### 2.5. Drugs and Materials

Semaglutide and liraglutide were purchased from MedChemExpress via the local distributor Hycultec (Beutelsbach, Germany). Exendin(9-39) was purchased from Thermoscientific via the local distributor Th. Geyer (Renningen, Germany). Exenatide was purchased from Toronto Research via the local distributor Biozol (Eching, Germany). Glucagon was purchased from Bachem (Bubendorf, Switzerland). Isoprenaline, nifedipine and H89 were purchased from Sigma Aldrich (Taufkirchen, Germany). Ryanodine and thapsigargin were purchased from Tocris via the local distributor Bio-Techne (Wiesbaden, Germany). All other chemicals (buffer compounds) were of the highest purity grade commercially available. Deionized water was used throughout the experiments to prepare the modified Tyrode’s solution. Stock solutions were prepared fresh daily.

## 3. Results

### 3.1. Mouse Atrial Preparations

**Figure 2:**
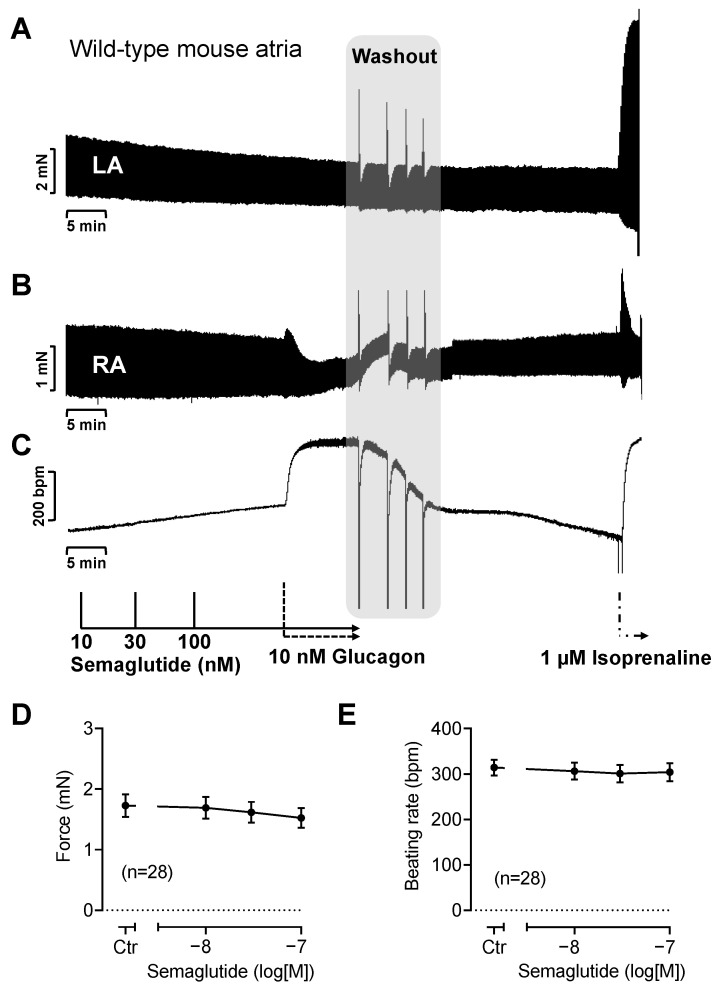
The effects of semaglutide in wild-type mouse atrial preparations. The original recordings of the effect of semaglutide on the FOC in isolated mouse left atrial (LA) preparations (**A**) or right atrial (RA) preparations (**B**). The effect of semaglutide on the beating rate in isolated spontaneously beating mouse right atrial preparations is shown in (**C**). Subsequently, glucagon and after complete drug removal (washout) a maximum effective concentration of isoprenaline (1 µM) were applied. The vertical bars in (**A**,**B**) indicate the FOC in millinewtons (mN) and in (**C**) the beating rate in beats per minute (bpm). Horizontal bars indicate time in minutes. (**D**) Summarized concentration–response curves for the effect of semaglutide on FOC in mouse left atrial preparations. (**E**) Summarized concentration–response curves for the effect of semaglutide on the beating rate in mouse right atrial preparations.

First, we studied the effects of semaglutide, liraglutide and exenatide in an animal model of the mammalian heart. Specifically, we interrogated whether semaglutide increased the FOC in electrically stimulated (1 Hz) left atrial mouse preparations, the FOC in spontaneously beating right atrial mouse preparations, and the beating rate in spontaneously beating right atrial mouse preparations. At concentrations up to 100 nM (the highest concentration studied), no significant positive inotropic or chronotropic effects of semaglutide were observed in the electrically stimulated left or spontaneously beating right mouse atrial preparations, respectively (Figure 2). As an additional control, we added 10 nM glucagon to the same left atrial and right atrial preparations from mice, in accordance with our previous findings [12]. Nevertheless, glucagon, similar to semaglutide, did not significantly alter the FOC in the mouse left atrium (Figure 2A). However, 10 nM glucagon increased the beating rate in spontaneously beating right atrial preparations (Figure 2C). Similar further experiments were quantified (Figure 2D,E). An amount of 1 µM of isoprenaline was used here as a positive control to increase force or beating rate. Isoprenaline acts via β-adrenoceptors and increased both beating rate (Figure 2C) and the FOC (Figure 2B) in right atrial preparations and the FOC in left atrial preparations (Figure 2A). The increase in the FOC by isoprenaline (and glucagon) in right atrial mouse preparations was only transient. This was probably due to the so-called “negative staircase” (“Treppe”) in the mouse atrium (e.g., [13]). When the beating rate increases, the Ca^2+^ handling in the mouse atrium is impaired and force gradually declines (Figure 2B). This could explain the transient positive inotropic effect of glucagon and isoprenaline in right atrial preparations (Figure 2A compared to Figure 2B). Of note, this is a species-dependent difference as a healthy isolated human atrium exhibits a “positive staircase” [14].

Like semaglutide, liraglutide and exenatide did not affect the FOC or beating rate in mouse atrial preparations.

### 3.2. Human Atrial Preparations

**Figure 3:**
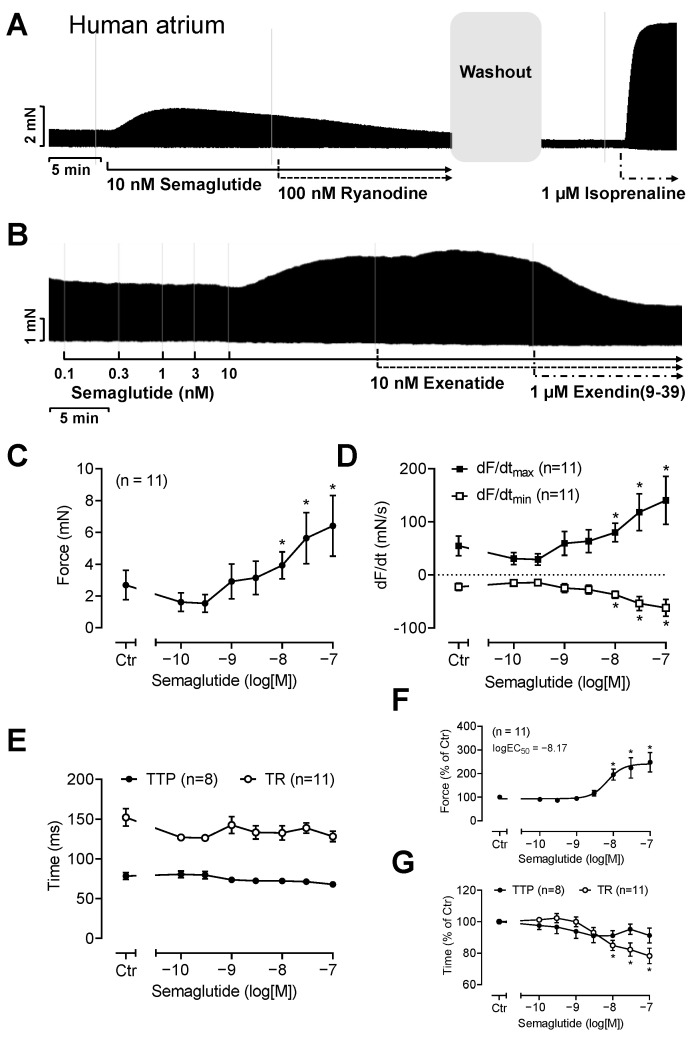
The effects of semaglutide in human atrial preparations (HAP). (**A**,**B**) Original recordings. (**A**) Semaglutide induced a time-dependent positive inotropic effect in HAP. Subsequently applied ryanodine (100 nM) reduced the positive inotropic effect of semaglutide and finally a maximum effective concentration of isoprenaline (1 µM) was applied to raise force of contraction. (**B**) In a separate experiment, semaglutide was cumulatively applied until 10 nM semaglutide had been added. Thereafter, exenatide (10 nM) was added followed by 1 µM of the GLP-1R antagonist exendin(9-39). Please note that the inotropic effect of semaglutide was completely reversed by exendin(9-39). Vertical bars in (**A**,**B**) indicate FOC in millinewtons (mN). Horizontal bars indicate time in minutes. Summarized concentration–response curves for the effect of semaglutide on FOC are presented in (**C**). Moreover, data are depicted on the effect of cumulatively applied semaglutide in HAP on the maximum rate of tension development (dF/dt_max_) and on the maximum rate of tension relaxation (dF/dt_min_) (**D**) as well as the time to peak tension (TTP) and time of tension relaxation (TR) (**E**). For comparison: in (**F**), FOC is presented as % of control (ctr = pre-drug value) and in (**G**), time parameters are presented as % of ctr. By normalization, the scattering between patient samples could be reduced. Abscissae indicate concentrations of semaglutide in the decadic logarithm of molar concentrations. The potency of semaglutide to increase FOC is given as logEC_50_. * *p* < 0.05 versus ctr. Numbers in brackets give the number of experiments.

This ineffectiveness of semaglutide to augment the FOC in electrically stimulated atrial preparations presents a further species difference: we noted positive inotropic responses to semaglutide in HAP (original tracings in Figure 3A,B). Gaining significance at 10 nM and up to 100 nM (the highest concentration studied), we detected a concentration- and time-dependent PIE of semaglutide in HAP. This effect is summarized in Figure 3C. Like isoprenaline, semaglutide increased the rate of tension development (Figure 3D), the rate of tension relaxation (Figure 3D) and reduced the muscle relaxation time (Figure 3E).

**Figure 4:**
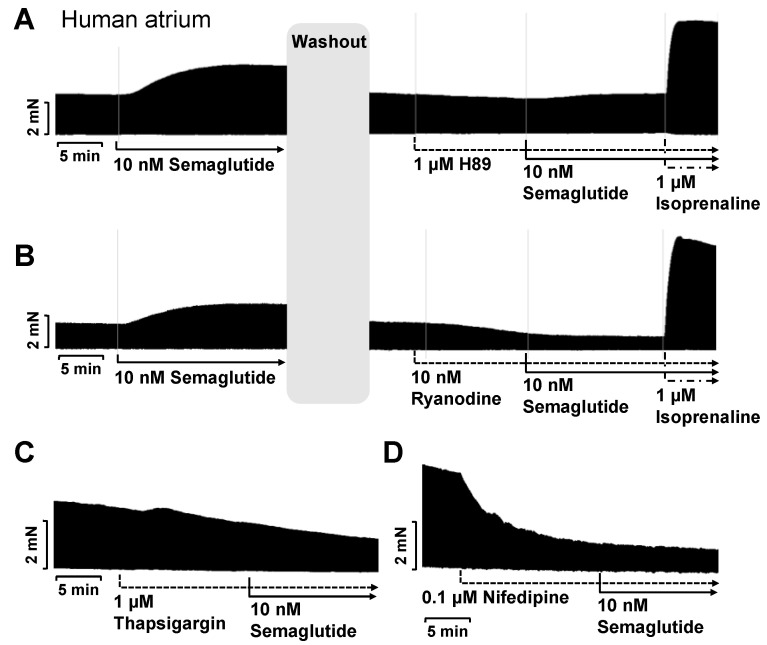
(**A**) In a typical separate experiment, 10 nM semaglutide elicited a positive inotropic effect. Then, a washout was performed. Thereafter, for the same muscle preparation, 1 µM of H89 was added, thereafter again 10 nM semaglutide and finally 1 µM isoprenaline. (**B**) In a further typical separate experiment, 10 nM semaglutide elicited a positive inotropic effect. Then, a washout was performed. Thereafter, to the same muscle preparation, 10 nM ryanodine was added, and thereafter again 10 nM semaglutide and finally 1 µM isoprenaline. Similarly, in (**C**), 1 µM thapsigargin and in (**D**), 0.1 µM nifedipine were applied prior to the addition of 10 nM semaglutide. The vertical bars indicate the FOC in millinewtons (mN). The horizontal bars indicate the time in minutes.

The positive inotropic effects of semaglutide in HAP were reversed when we added the GLP-1R antagonist exendin(9-39) (Figure 3B). In separate experiments, we non-cumulatively applied only 10 nM semaglutide (Figure 3A and Figure 4). We studied 10 nM semaglutide, because this is a typical concentration reached in clinical studies [15]. First, 10 nM semaglutide was applied to establish a PIE. Then, the PIE was washed out by repeated changes of the buffer in the organ bath. Then, we applied 1 µM H89 (Figure 4A) or 10 nM ryanodine (Figure 4B). These drugs attenuated the positive inotropic effects of subsequently applied semaglutide (Figure 4). Moreover, 1 µM thapsigargin (Figure 4C) and 0.1 µM nifedipine (with 1 µM nifedipine the muscles ceased to beat) were applied (Figure 4D). For instance, under these conditions, 10 nM semaglutide alone increased the FOC to 162 ± 18% (n = 15, *p* < 0.05). But in the presence of 1 µM thapsigargin, 10 nM ryanodine, 1 µM H89 or 0.1 µM nifedipine, 10 nM semaglutide increased force by only 70 ± 11%, 52 ± 7%, 115 ± 11% and 56 ± 11% (n = 3–5, *p* < 0.05 vs. semaglutide alone), respectively.

**Figure 5:**
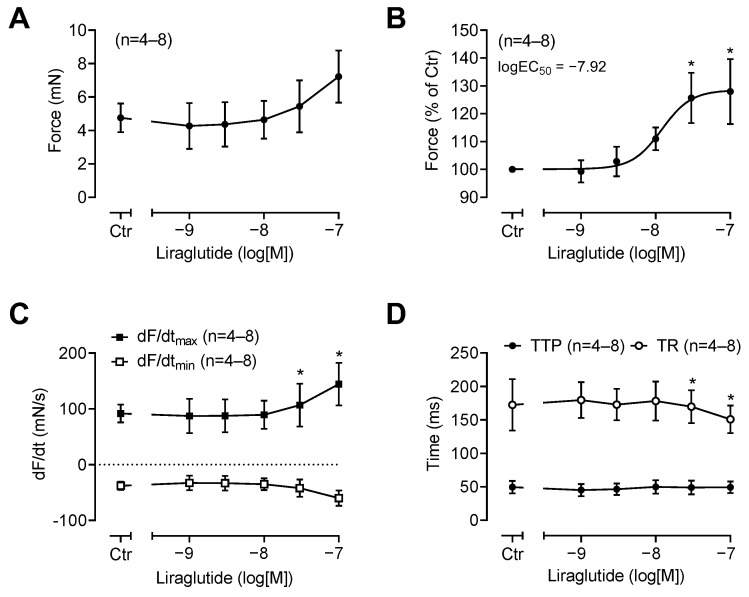
Summarized concentration–response curves for the effect of liraglutide on FOC in millinewtons (mNs) (**A**) or as % of control (ctr = pre-drug value) (**B**), the maximum rate of tension development (dF/dtmax) and maximum rate of tension relaxation (dF/dtmin) (**C**) as well as the time to peak tension (TTP) and time of tension relaxation (TR) in milliseconds (ms) (**D**). Abscissae indicate concentrations of liraglutide in the decadic logarithm of molar concentrations. The potency of liraglutide to increase FOC is given as logEC_50_. * *p* < 0.05 versus ctr. Numbers in brackets give the number of experiments.

Similarly, as for semaglutide, starting at 30 nM, we detected a PIE of liraglutide in HAP. These data are summarized in Figure 5A,B. Like for semaglutide, this PIE of liraglutide was accompanied by an increase in the rate of tension development and in the rate of tension relaxation (Figure 5C) and by a reduction in muscle relaxation time (Figure 5D).

**Figure 6:**
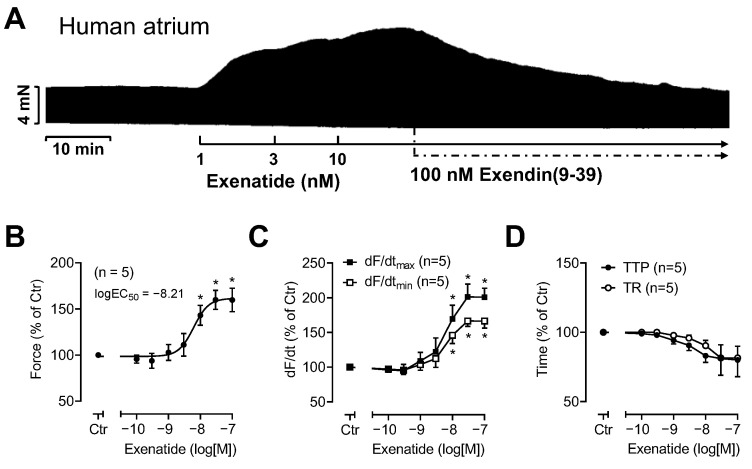
(**A**) An original recording of the concentration- and time-dependent positive inotropic effect of exenatide in millinewtons (mN) in HAP. After 10 nM exenatide had been given, 100 nM exendin(9-39) reduced the FOC in HAP. Horizontal bars indicate the time axis in minutes (min). (**B**–**D**) Summarized concentration–response curves for the effect of exenatide on the FOC (**B**), the maximum rate of tension development (dF/dtmax) and tension relaxation (dF/dtmin) (**C**) as well as the time to peak tension (TTP) and time of tension relaxation (TR) (**D**) all as % of control (ctr = pre-drug value). Abscissae indicate concentrations of exenatide in the decadic logarithm of molar concentrations. The potency of exenatide to increase FOC is given as logEC_50_. * *p* < 0.05 versus ctr. Numbers in brackets give the number of experiments.

For comparison, we studied exenatide, because exenatide had raised the FOC in HAP in work by others [2]. Exenatide increased the FOC in the HAP in a concentration- and time-dependent manner (original recording in Figure 6A, summarized in Figure 6B). In other words, with more time passing since the addition of exenatide, more force was generated and higher concentrations of exenatide raised force more than lower concentrations. These dependencies are consistent with causality. Subsequently added exendin(9-39) abolished the PIE of exenatide (Figure 6A), confirming a previous publication by others [2]. As a new finding we noted that, like semaglutide or liraglutide, exenatide also increased the rate of tension development, as summarized in Figure 6C. Moreover, exenatide shortened the time of relaxation (Figure 6D).

**Figure 7:**
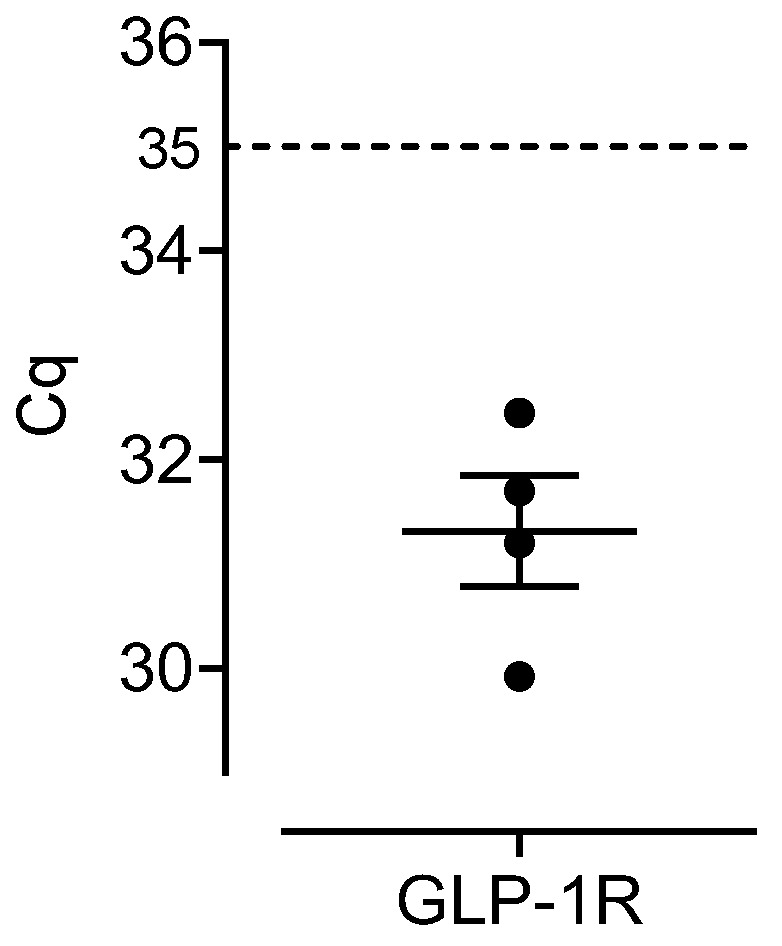
The gene expression of glucagon-like peptide 1 receptor (GLP-1R) in the right atrium of the human heart. Data are presented as individual Cq values for each measured sample. The detection limit was set to Cq = 35 and therefore Cq values lower than 35 (Cq < 35) were considered as expressed in the tissue.

The effects of semaglutide in the human atrium could be mediated by GLP-1R: we could observe the mRNA expression of the GLP-1R in human right atrial tissue from four patients (Figure 7).

## 4. Discussion

The most important new findings of this paper are that semaglutide and liraglutide induced a PIE in the isolated human atrium at concentrations achieved in human plasma at therapeutic doses. Hence, the PIE could be clinically relevant. These effects of semaglutide and liraglutide are probably mediated via GLP-1R because they are antagonized by exendin(9-39), an antagonist at GLP-1R. The GLP-1R signal is probably mediated intracellularly via cAMP, as this is the general signal transduction of this receptor in cell culture [2,16]. Further evidence for an involvement of cAMP in the PIE of semaglutide comes from our observation that H89, an inhibitor of the activity of the cAMP-dependent protein kinases, is also used in studies of exenatide [2], and attenuated the PIE of semaglutide.

Semaglutide and liraglutide can act via GLP-1R, because these receptors are present in the human atrium [2,17,18]. We confirmed this finding (see Figure 7).

Moreover, phosphorylations due to the cAMP-dependent protein kinases should have led to alterations in Ca^2+^-homeostasis in the heart (Figure 1). We inferred this from the fact that the PIE of semaglutide was attenuated by ryanodine, thapsigargin and nifedipine. These drugs inhibit the release of Ca^2+^ from ryanodine receptors in the junctional sarcoplasmic reticulum, the uptake of Ca^2+^ by SERCA in the free sarcoplasmic reticulum and the Ca^2+^ current through the L-type calcium channel in the sarcolemma (Figure 1).

GLP-1 binds to the human GLP-1R with an affinity of around 5 nM [16]. Hence, our concentration–response curves were closely aligned to these biochemical data.

An atrial and ventricular effect of GLP-1R agonism in the human heart has been reported before by others. In isolated electrically driven atrial muscle preparations from ten patients, the selective GLP-1 receptor agonist exenatide (at 6 nM, the highest concentration studied) raised the FOC in a concentration- and time-dependent manner [2]. In only two of 14 ventricular muscle strips (i.e., about only 14% of patients) from non-failing human (donor) hearts, exenatide exerted a PIE. In contrast, all human atrial and human ventricular samples in their study expressed the mRNA of the GLP-1R [2]. We could confirm and extend their findings in HAP: exenatide as reported raised FOC; however, semaglutide, as expected for a cAMP-increasing agent and well known for the prototypical agonist isoprenaline, increased tension rate parameters and reduced tension time parameters. These latter effects are well understood as a consequence of the increased phosphorylation of regulatory proteins in HAP.

### 4.1. Species Differences

Semaglutide, liraglutide and exenatide had no PIE in the isolated mouse left atrium (this study). This finding is new but plausible from published data. GLP-1R mRNA was highest in the mouse right atrium, much lower in the mouse left atrium, and missing in both the mouse left and right ventricle [18,19]. One could therefore simply argue that the reduced expression of GLP-1R mRNA in the mouse left atrium compared to the mouse right atrium could explain why we could not detect a positive inotropic effect in the mouse left atrium. However, cellular heterogeneity might play an additional role here: Ali et al. [19] measured expression in total tissue homogenates from the mouse cardiac regions. Thus, they could not discern the cell type where the receptor was located. Indeed, the same group later reported that GLP-1R seems to be confined in its expression to the mouse heart’s endothelial cells [20]. Then, the question can be asked why we measured a positive chronotropic effect in mouse right atrial preparations that were beating spontaneously. In our view, this could mean that the expression of GLP-1R is restricted to sinus node cells in the mouse heart: their number might be too low to detect with current methods.

In contrast, in the human atrium, GLP-1R was mainly, if not exclusively, detected as mRNA in cardiomyocytes [20]. In situ hybridization detected mRNA for GLP-1R in human sinoatrial cardiomyocytes, further suggesting a physiological role of the GLP-1R in heart rate regulation in humans [17].

In isolated adult rat ventricular cardiomyocytes, the stimulation of GLP-1R increased cAMP levels but reduced contractility. This reduced contractility was accompanied by and explained by a reduction in the pH in the isolated ventricular rat cardiomyocytes and the authors also measured a desensitization of the myofilaments to Ca^2+^ in the presence of GLP-1 [21]. In isolated perfused rat hearts, conflicting results were reported. In one study GLP-1 was given to isolated adult rat hearts combined with a protease inhibitor (to assure the intactness of GLP-1). However, the authors noted no change in left ventricular pressure or heart rate in their rat Langendorff hearts [22]. However, other studies used GLP-1 (7-36, an active metabolite of GLP-1) without protease inhibitors and found a reduction in left ventricular pressure but no change in heart rate in isolated rat hearts [23]. Very recently, GLP-1 was reported to increase the beating rate in isolated adult rat right atrial preparations [24]. The positive chronotropic effects (PCEs) of 100 nM GLP-1(7-39) were blocked by 100 nM exendin(9-39), by 10 µM indometacin or by 100 µM L-NAME, an inhibitor of the activity of nitric oxide synthases. These PCEs of GLP-1 were assumed to be mediated by GLP-1R through phospholipase A2 and nitric oxide synthase [24]. This discrepancy in rat studies might stem from methodological differences between the laboratories involved.

As concerns the mouse heart, there are also some differences in the literature which are apparent: Liraglutide elevated cAMP levels in neonatal ventricular mouse cardiomyocytes (in the presence of the unspecific phosphodiesterase inhibitor 3-isobutyl-1-methylxanthine, 100 µM; incubation for 30 min with 100 nM liraglutide; blocked by 10 µM exendin(9-39) [25]) and in isolated adult mouse atrial cardiomyocytes (in the presence of the unspecific phosphodiesterase inhibitor 3-isobutyl-1-methylxanthine, 100 µM; incubation for 30 min with 128 nM liraglutide [26]). However, liraglutide (30 nM) failed to increase left intraventricular pressure in isolated perfused adult mice [26]. And liraglutide failed to increase cAMP levels in atrial cardiomyocytes from mice with GLP-1R knockout [26]. This finding indicates that cAMP was elevated via GLP-1R in wild-type cardiomyocytes. The latter finding is puzzling because the same group later described how, in the adult mouse heart, only endothelial cells express GLP-1R [20]. Hence, there must be methodological discrepancies between these conflicting studies in mice. The cell cultures of cardiomyocytes might have contained unwittingly contaminating endothelial cells in which the cAMP was raised, which led to an increase in cAMP in the whole sample. Liraglutide failed to increase the beating rate in isolated adult mouse right atrial preparations (this study), this in agreement with a previous publication, that used a somewhat different methods compared to the present study [18]. In contrast, in isolated adult mouse sinoatrial cardiomyocytes, 100 nM liraglutide increased the spontaneously beating rate [27]. However, Baggio et al. [18], in contrast to the present study, failed to include data on the inotropic effects of liraglutide on isolated right or left mouse atrial preparations, which we report here as an extension to their work. A perfusion of isolated adult mice hearts with 0.5 nM GLP-1 or 5 nM lixisenatide (a GLP-1R agonist) did not alter left ventricular pressure or heart rate [18]. This suggests that the GLP-1 receptor is absent in the mouse heart (in the sinus node and the left ventricle) or that the concentrations used of GLP-1 or 5 nM lixisenatide were too low in order to stimulate these receptors or that the signal transduction does not follow expected patterns.

In isolated cardiomyocytes from adult canine left ventricles, GLP-1 at 5 nM increased the current through the L-type calcium channel by about 23% and prolonged the duration of the action potential [28]. These effects were blocked by preincubation of the adult canine cardiomyocytes with the GLP-1R antagonist exendin(9-39) [28].

### 4.2. Clinical Relevance

In an extensive meta-analysis comprising >70,000 patients, GLP-1R agonists were usually not found to increase the risk of arrhythmia in diabetic patients. Only at high doses in patients with a high body mass index (BMI) was there a risk for ventricular arrhythmias [29]. In contrast, Neves et al. [30] detected in a post hoc analysis that, at a minimum, liraglutide tended to worsen heart failure with reduced ejection fraction (HFrEF) and to increase the incidence of total arrhythmias in patients.

It is unlikely that the PIE of liraglutide or semaglutide has a beneficial effect on heart failure. The GLP-1R is seldom (in about 14% of healthy humans) functional in the human ventricle [2]. Hence, beneficial ventricular effects should rarely occur. However, in each and every patient, one would expect a PIE of semaglutide in the human right atrium. This might be helpful in some cases of heart failure. It can be argued (see [2] for extended discussion) that when force in right human atrial preparations in vitro is enhanced, then contractility in the left atrium should also probably increase as both atria show expression of GLP-1 receptors and usually respond similarly to drugs. If this were the case, the left atrium would beat more vigorously and thus pump more blood into the left ventricle per unit of time. A higher filling of the left ventricle should lead to a higher ejection fraction and thus better blood flow to the dependent organs, which could be helpful for some patients with heart failure, at least those who do not suffer from HFrEF.

### 4.3. Limitations of the Study

We have not directly studied the effects of semaglutide on the sinus node of humans and we have not measured the effects of semaglutide on contractility in human ventricular tissue. Antibodies that detect human cardiac GLP-1R are currently not available.

## 5. Conclusions

In conclusion, we present evidence for a positive inotropic effect of semaglutide via GLP-1 receptors in human atrial preparations.

## Data Availability

The raw data supporting the conclusions of this article will be made available by the authors on request.
